# Modeling of replicative senescence in hematopoietic development

**DOI:** 10.18632/aging.100072

**Published:** 2009-07-23

**Authors:** Anna Marciniak-Czochra, Thomas Stiehl, Wolfgang Wagner

**Affiliations:** ^1^ Interdisciplinary Center of Scientific Computing (IWR), Institute of Applied Mathematics, University of Heidelberg, 69120 Heidelberg; ^2^ Heidelberg Academy of Sciences and Humanities, 69117 Heidelberg, Germany; ^3^ Helmholtz Institute for Biomedical Engineering, Aachen University Medical School, 52074 Aachen, Germany; ^4^ Department of Medicine V, University of Heidelberg, 69120 Heidelberg, Germany

**Keywords:** mathematical model, hematopoietic stem cells, aging, replicative senescence, self-renewal

## Abstract

Hematopoietic stem cells (HSC) give rise to an enormous number of blood cells throughout our life. In contrast their number of cell divisions preceding senescence is limited underin vitro culture conditions. Here we consider the question whether HSC can rejuvenate indefinitely or if the number of cell divisions is restricted. We have developed a multi-compartmental model for hematopoietic differentiation based on ordinary differential equations. The model is based on the hypothesis that in each step of maturation, the percentage of self-renewal versus differentiation is regulated by a single external feedback mechanism. We simulate the model under the assumption that hematopoietic differentiation precedes the six steps of maturation and the cells ultimately cease to proliferate after 50 divisions. Our results demonstrate that it is conceivable to maintain hematopoiesis over a life-time if HSC have a slow division rate and a high self-renewal rate. With age, the feedback signal increases and this enhances self-renewal, which results in the increase of the number of stem and progenitor cells. This study demonstrates that replicative senescence is compatible with life-long hematopoiesis and that model predictions are in line with experimental observations. Thus, HSC might not divide indefinitely with potentially important clinical implications.

## Introduction

Human and animal tissues are continuously renewed by somatic stem cells. A decline of stem cell function will inevitably impair the regenerative potential and result in the aging process of the organism [1]. Hematopoietic stem cells (HSC) give rise to all lineages of blood cells. At least some of their progeny has to retain stem cell function in order to maintain the stem cell pool. It is still controversial if this self-renewal with regard to the differentiation potential also implies that HSC can rejuvenate indefinitely or if they are destined to age as other somatic cells do [2]. This might also have im-plications for HSC transplantation:  A child that receives an allogeneic transplant from an elderly donor will eventually have a hematopoietic system of age that exceeds the expected maximal age of humans. Under normal conditions, there are no signs of anemia in elderly people although the capacity for hematopoietic recovery under stress conditions appears to gradually decline [3]. Aging affects the immune system and the number of lymphocytes significantly decreases [4]. Transplantation experiments indicated that this myeloid skewing of differentiation potential with age is due to intrinsic changes in older HSC [5,6].

**Figure 1.**
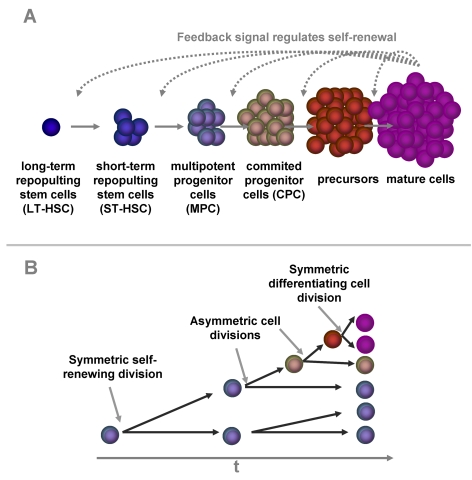
Self-renewal and differentiation in hematopoiesis. Hematopoietic differentiation is a multi-step process. A small group of long term repopulating hematopoietic stem cells (LT-HSC) replicates very slowly. The down-stream compartments are more and more committed to a specific linage and replicate at faster rates. Some of the progeny have to self-renew to keep the pool of hematopoietic stem and progenitor cells. Our model is based on the hypothesis this percentage of self-renewal *versus* differentiation is regulated by a feedback mechanism that is related to the number of mature cells in the blood (**A**). There is evidence, that the dual function of self-renewal and differentiation is regulated by asymmetric cell divisions where one daughter cell retains the stem cell function whereas the other differentiating cell becomes a faster proliferating precursor cell. Alternatively, cells can undergo symmetric cell divisions to produce either two identical, self-renewing cells or two differentiated daughter cells (**B**).

Hematopoiesis is a multi-step process, in which a relatively small population of HSC gives rise to all types of blood cells. The current understanding is based on a hierarchical tree in which each lineage of blood cells proceeds through a chain of maturation stages, which are sequentially traversed. At least six different compartments have been proposed although so far experimental data do not provide a precise distinction between these stages (Figure 1A): 1) A small subset of HSC is capable of long-term repopulation upon transplantation (LT-HSC). These cells can be enriched by their immunophenotype as CD34^+^CD38^-^CD90^+^ and they are usually quiescent or very slow dividing [7-11]. 2) The next compartment comprises short-term repopulating stem cells (ST-HSC) that sustain hematopoiesis only for a limited time of several weeks or months after transplantation and these may correspond to a CD34^+^CD38^-^ phenotype [12]. 3) Multipotent progenitors cells (MPC; such as common myeloid progenitor cells) are included in the CD34^+^CD38^+ ^cell fraction. In the later stages CD34 expression is absent and lineage specific markers are expressed. There is evidence that maturation proceeds via 4) committed progenitor cells (CPC; such as the granulocyte-macrophage colony-forming cells), 5) precursor cells with single-lineage potential (such as the granulocyte-progenitors) and ultimately 6) mature cells with a limited lifetime (such as granulocytes). Upon every cell division, some progeny cells have to maintain the stem cell pool (self-renewal), whereas the others proceed to the next maturation compartment (differentiation). There is evidence, that the relation between self-renewal and differentiation is controlled by asymmetric cell divisions and this correlates with asymmetric cell division kinetics of the progeny cells (Figure 1B) [13-15].

Research on aging in adult stem cells is limited by the methods available for their identification, purification and culture expansion. However, all other cell types, including hematopoietic cells, fibroblasts and mesenchymal stromal cells enter a senescent state after a certain number of cell divisions. Within about 30 to 50 population doublings, the cells enlarge and become more granular with an irregular cell shape [16,17]. Ultimately they irrevocably stop dividing although they remain metabolically active and can be maintained in this state for years. It has been demonstrated that similar cell enlargement can also be induced under growth stimulation when the cell cycle is blocked: cells senesce if expression of p21 is induced ectopically and this is accompanied by beta-galactosidase staining, cellular hypertrophy, increased levels of cyclin D1 and active TOR (target of rapamycin, also known as mTOR) [18]. Notably, the loss of proliferative potential can be decelerated by rapamycine indicating that senescence can be pharmacologically suppressed [19].

The phenomenon of replicative senescencewas first described in the 1960s by Leonard Hayflick [20]. Since then, it is debated if reaching the so-called "Hayflick limit" might be related to the aging of the whole organism.

Human hematopoietic system accounts for an estimated output of more than 10^11^ cells per day and approximately 4 x 10^15^ cells over a life time [21,22]. In theory this cell number could be reached from an individual cell by 52 cell divisions but the question remains if replicative senescence is really compatible with sustained hematopoiesis and how this affects the relationship of stem cells with their more differentiated counterparts.

In our previous work, we have described multi-compartment models to investigate possible mecha-nisms of regulation and stabilization of blood cell production, following perturbations such as bone marrow transplantation. We have demonstrated using mathematical modeling that feedback control of the frequency of self-renewal of HSC is most essential for ematopoietic reconstitution following transplantation [23]. This model was now adapted to determine the effect of replicative senescence on hematopoietic development. The aim of this study was to determine if a limitation to 50 cell divisions is compatible with hematopoiesis over an entire life-span and how this affects the ratio of stem cells number to that of their differentiated progeny.

**Figure 2.**
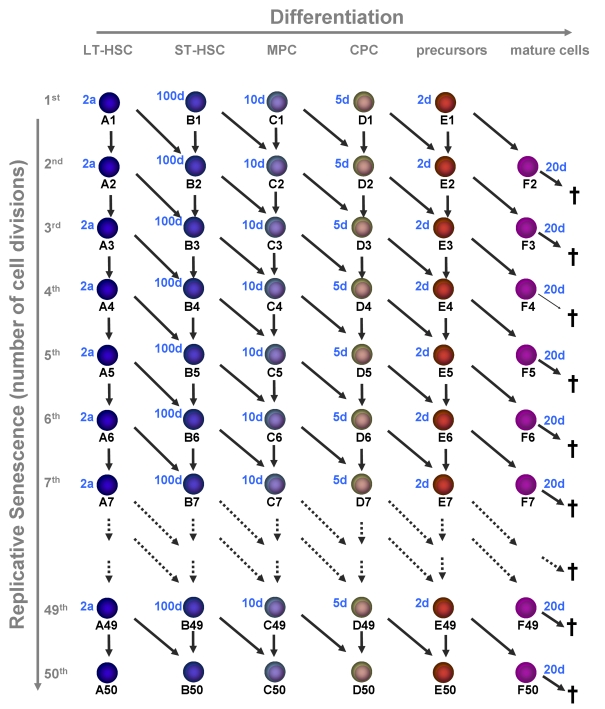
Replicative senescence in hematopoietic development. In this model we have addressed the question if hematopoiesis is compatible with a restriction in cell divisions (e.g. 50 cell divisions). Upon each division the daughter cells may either remain on the same maturation level or proceed to the next step of differentiation. Proliferation rates increase upon differentiation and the estimated times are indicated for each maturation step. Mature cells are post-mitotic and die after 20 days.

**Figure 3.**
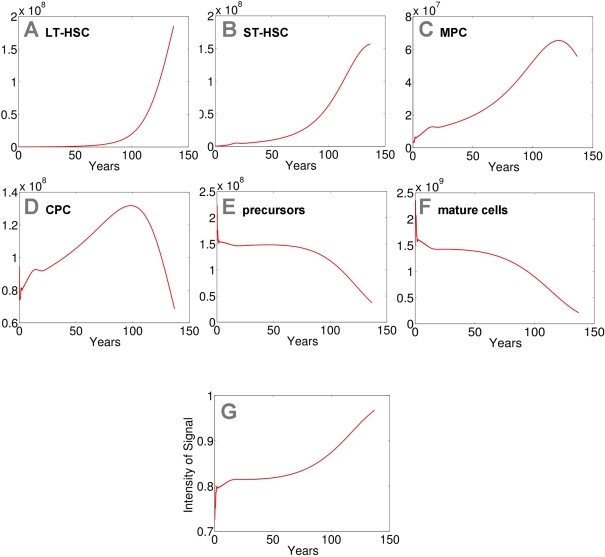
Modeling of replicative senescence in hematopoiesis. Cell numbers of the different compartments are plotted over a time course of 140 years (A: LT-HSC; B: ST-HSC; C: MPC; D: CPC; E: precursors; F: mature cells). The plots A-F depict the dynamics of the cells. In addition the progression of the signal is demonstrated (G). Input cell numbers were chosen close to the local equilibrium. Our model demonstrates that, under the assumptions on model parameters, hematopoiesis can be maintained for more than 100 years with a restriction to 50 cell divisions. However, the number of mature cells declines over time and the feedback signal increases correspondingly. Therefore the percentage of self-renewal increases resulting in a higher number of stem cells and progenitor cells.

## Results

We have developed a mathematical model describing the dynamics of cells of *n* different differentiation stages under the restriction that the number of possible cell divisions is limited by an arbitrary number *m* (see Methods for the details of the model). We have solved numerically the model with *n=6* stages of maturation (long-term repopulating stem cells (LT-HSC), short-term repopulating stem cells (ST-HSC), multipotent progenitor cells (MPC), committed progenitor cells (CPC), precursors, and mature cells) and*m=50* possible cell divisions (Figure 2). We assume that, neither proliferation rates nor self-renewal fractions, depend on the number of cell generation but they only depend on the stage of differentiation. For the initial cell numbers at birth (0 years) we have chosen cell numbers close to the steady states value of the model without replicative senescence  (LT-HSC = 10^5^; ST-HSC = 5x10^5^; MPC = 4 x 10^6^; CPC = 8 x 10^7^; precursors = 1.5 x 10^8^; mature cells = 1.5 x 10^9^).

As anticipated a prerequisite for this model is that LT-HSC are very slow dividing. Otherwise, the stem cell pool is rapidly depleted. This might explain why ST-HSC cannot maintain hematopoiesis for a long time. We have chosen a proliferation rate of LT-HSC of once every two years with a maximum number of 50 cell divisions to adapt the results to a human life-time. Furthermore, the rate of HSC self-renewal (a_1_) has to be larger than the corresponding rates for the other compartments (*a_1_>a_i_* for *i=2,3,4,5*). Otherwise, the compartment with the highest self-renewal potential takes over the stem cell function, whereas all up-stream compartments including the HSC compartment eventually become extinct. This has also been described in our previous work. For numerical simulations we have chosen the same maximal self-renewal rates as in our previous work [23] (LT-HSC = 0.7; ST-HSC = 0.65; MPC = 0.65; CPC = 0.65; precursors = 0.55; mature cells do not divide). Under these assumptions our model demonstrates that hematopoiesis can be sustained over a life-time with only 50 cell divisions. However, hemato-poiesis does not reach a steady state. The number of mature cells slowly declines after about 50 years and after 140 years hematopoiesis ceases to take place.

Loss of mature cells is partly compensated by an increased feedback signal that enhances the self-renewal rates. Hence, the number of mature cells inversely correlates with the intensity of the signal that increases over the years as some of the cells in the stem cell fraction reach the Hayflick limit. Therefore, other than anticipated, the number of stem and progenitor cells increases with aging. Hence, elderly people have a higher number of stem cells whereas their remaining number of cell divisions is more restricted (Figure 3).

**Figure 4.**
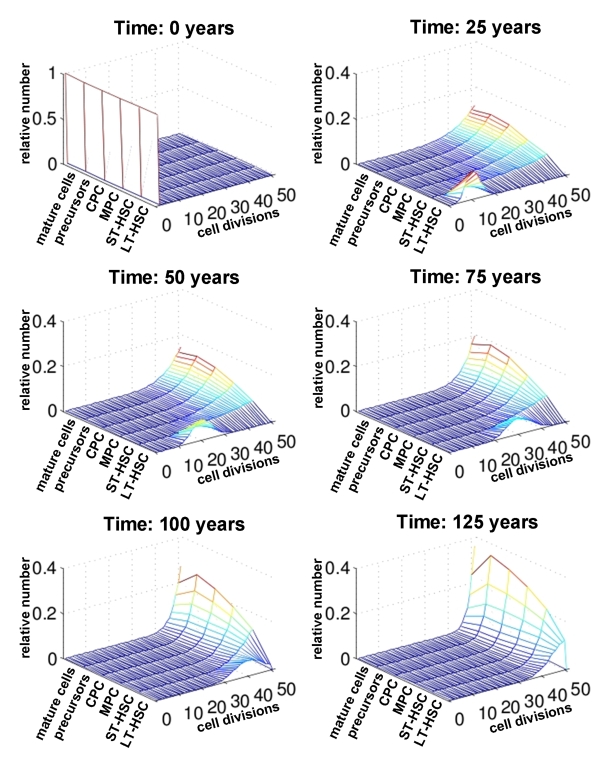
Number of cell divisions over time. For each compartment of differentiation the relative number of cells is plotted against the number of cell divisions (0 to 50). The distribution is compared at different time points (0, 25, 50, 75 and 100 years). This indicates that changes upon aging are more prominent in the stem cell compartment than in mature cells.

Subsequently, we have analyzed the distribution of the number of cell divisions for each maturation step at different ages (0 years, 25 years, 50 years, 75 years, 100 years and 125 years; Figure 4). Under the assumptions of our model all cells can undergo 50 cell divisions counting from the time of birth of the individual. As expected, the number of cell divisions in the LT-HSC compartment decreases continuously with age. However, for the more differentiated compartments the majority of cells already performed more than 45 cell divisions after only a few years. Hence, the effects of replicative senescence upon aging are most obvious in the stem cell compartment. Therefore, age-associated changes should be observed in adult stem cells rather than their differentiated progeny.

## Discussion

This study demonstrates that a limitation of the number of cell divisions is consistent with normal human hematopoiesis. Furthermore, the system of cells undergoing replicative senescence does not necessarily have a positive steady state. During aging the feedback signal increases to sustain the number of mature cells. Hence, the quantity of progenitor cells increases whereas their quality with regard to long-term proliferation decreases. The model presented here does not distinguish between different lineages of differen-tiated blood cells precursors, a simplification, which assumes that all the lineages have the same proliferation and maturation structure and dynamics. Also the numbers of compartments and the maximum number of cell divisions have been arbitrarily chosen. It might be speculated, that the proliferation rate decreases with the increase in the number of cell divisions. For simplicity this has not been considered in our model but it would result in a similar reduction of the regenerative potential.

Other models have described aging of differentiated epithelial tissue as the consequence of replicative senescence of progenitor cells only [24,25]. Further-more, it has been suggested that hematopoiesis proceeds through a significantly higher number of different compartments and that about 31 mitotic events separate the HSC from the mature cells [26]. To our knowledge, the present study provides the first model that treats replicative senescence and maturation of stem cells as two independent processes. It is based on the assumption that stem cells are quiescent or very slow dividing. Indeed, it is commonly accepted, that LT-HSC are quiescent or very slow dividing and that they can be enriched by slow division kinetics [8,10,11,14,15]. Recently, Wilson and co-workers have demonstrated that there was a dormant-fraction of HSC that divided only five times during the lifetime of mice and especially that these dormant HSC display repopulating activity following a serial transplantation [9]. For the human system there are no clear biological data available for the proliferation rate of LT-HSC or for the number of maximum cell divisions. Clearly, slow divisions kinetics reduce the risk of mutagenesis and defects during cell division [27]. These slow division kinetics are also a prerequisite to maintain hemato-poiesis in our model. Otherwise the stem cell pool might rapidly be depleted, even if a significantly higher number of population doublings was assumed.

In the murine transplantation model the potential for engraftment decreases after serial transplantations. The reconstituting ability declines continuously within 4 to 5 transfers [28,29]. Various studies have indicated that the functional ability of HSC in the repopulation model significantly declines with an increasing donor age [30]. It has been suggested that HSC from older mice have a significantly lower cycling activity than those isolated from younger mice [31]. There are several differences between different mouse strains and it needs to be verified if observation from the murine system can be extrapolated to humans. However, the data indicate that there are cell intrinsic changes in stem and progenitor cells during aging. At least some of these changes might be attributed to a lower number of remaining cell divisions.

Furthermore, competitive repopulation assays in murine transplantation models demonstrated that bone marrow of older mice had a higher number of ST-HSC and progenitor cells [32,33]. This is in line with similar *in vitro* experiments using the cobblestone area forming cell (CAFC) assay as a surrogate assay for primitive progenitor cells that demonstrated that their number increased about four-fold with age [6,34,35]. Initially this was not expected, as it has been anticipated that aging may be caused by the depletion of stem and progenitor cells. Interestingly, this increase of stem and progenitor cells also manifests in this study: during aging the number of mature cells declines slightly and correspondingly the feedback signal for self-renewal increases. Therefore, the number of stem and progenitor cells increases during aging.

The molecular mechanisms that trigger aging or senescence of HSC are still unknown. Shortening of telomeres was proposed as a biological clock that determines the number of cell replications. The idea of telomere erosion after about 50 cell divisions might be easily introduced in into this model. In fact, there have been reports that telomeres in HSC from bone marrow and peripheral blood are shorter than in those from peripheral blood [36]. There have also been reports that the length of telomeres decreases as a function of age [22]. We have analyzed telomere length in human CD34^+^ HPC and there was a tendency for shortening of telomeres with age although it was not significant [37]. There is increasing evidence, that progressive shorten-ing of the telomeres is not the only underlying mechanism and that it might represent an effect rather than the cause of aging [38-40]. Other causal molecular events and stochastic mechanisms also are compatible with this model. It has been suggested that senescence is triggered e.g. by DNA damage, accumulation of the cyclin-dependent kinase inhibitor p16INK4a or oxidative stress [1,41,42]. Alternatively, aging of HSC might be influenced by the cellular microenvironment in the bone marrow - the so called stem cell niche [2,43]. We have demonstrated that replicative senescence of mesenchymal stromal cells (MSC) affects their hematopoieisis supportive function [44]. By regulation of the proliferation rate and maintenance of HSC in a quiescent state the stem cell niche would play a central role in counteracting the replicative senes-cence.

Recently, we have described gene expression changes in CD34^+^ hematopoietic progenitor cells (HPC) from healthy donors of different age (0 years to 73 years). Various genes revealed significant gene expression changes indicating that our stem and progenitor cells are not protected from aging [37]. Interestingly, these changes are related to gene expression changes displayed in long-term culture of MSC from human bone marrow [17]. The concordance of age-related changes in HPC and of replicative senescence in MSC provides further evidence that our stem and progenitor cells undergo a similar process also *in vivo*.

Our model demonstrates that replicative senescence in the hematopoietic system is conceivable, and the results are compatible with various observations such as i) slow proliferation rate of HSC, ii) cell-intrinsic changes during aging, and iii) increasing number of stem and progenitor cells. Therefore, the possibility that the number of cell divisions for stem and progenitor cells is restricted requires careful consideration. It might have implications for the proliferative stress, such as after chemotherapy and for the long-term performance after stem cell transplantation with transplants from elderly donors.

## Methods

Derivation of the mathematical model. The model presented in this paper is a modified version of the discrete compartmental model with feedback (henceforth called the DCF model) proposed in [23]. The novelty of the model presented here is the assumption that replicative senescence distinguishes among subpopulations of different generations (DCF model with senescence). The model is based on two major assumptions: 1) in analogy to the DCF model, hematopoiesis is considered as a process during which cells traverse a finite number of subsequent discrete stages of differentiation and 2) it is assumed that each cell is able to perform a fixed finite number of cell divisions before it loses the ability to divide. In general we consider n differentiation stages and mdivisions that a cell can undergo.

As in the DCF models behaviour of each cell type is described by three parameters: 1) proliferation rate describing how often, on the average, a cell divides per unit of time; 2) the fractions of self-renewal and differentiation during cell division and 3) the death rate equal to the fractions of a specified cell subpopulation which dies per unit of time.

Differentiation and Senescence represent two indepen-dent dimensions. The model describes the following scenario: After division a cell gives rise to two progeny cells. Cell divisions can be symmetric or asymmetric. Therefore, we assume that on the average the fraction a of progeny cells remains at the same stage of differentiation as the parent cell, while the 1 - a fraction of the progeny cells differentiate, i.e. transfers to the higher differentiation stage. To account for the finite number of cell divisions (equal to m) we divide each stage of differentiation into m partitions. The partitions are numbered from 0 to m and the index of the partition indicates how many divisions the cells performed since the starting time t = 0. We refer to this number as a generation number. Thus, the progeny cells always belong to the next generation compared tothe parent cell, independently from their differentiation fate after division.

Treating the cell cycle as a well-mixed tank, the scenario described can be modeled by a system of *m + 1* times *n* ordinary differential equations (ODEs). Denote by *c_i.j_(t)* (*1 ≤  i≤ n, 0 ≤ j ≤ m*) the size of the *j*^th^ partition of the *i* stage of differentiation at time *t*, i.e., the amount of cells which belong at time *t* to the *i*^th^ stage of differentiation and have performed *j* divisions since the starting time t=0. Denote the proliferation rate of the subpopulation *c_i,j_* at time *t* by *p_i,j_(t)*, the fraction (probability) of self-renewal by *a_i,j_(t)* and the death rate by *d(t)*. In the following the time evolution $\frac{dc_{i,j}(t)}{dt}$ of *c_i,j_* is described.

Modeling of primitive stem cells. Starting with c_1,0_ ; the flux to mitosis at time t is given by p_1,0_(t)c_1,0_(t) and the flux to cell death is given by d_1,0_(t)c_1,0_(t). Since c_1,0_ denotes the number of stem cells that have divided *0* times, there exists no influx to this compartment.  Therefore

_$\frac{dc_{1,0}(t)}{dt}=-(d_{1,0}(t)+p_{1,0}(t))c_{1,0}(t)$_.

For *c_1,j_*, 0 < j < m; there exists additionally the influx to this compartment given by *2a_1,j-1_(t)p_1,j-1_(t)c_1,j-1_(t)*. Here *p_1,j-1_(t)c_1,j-1_(t)* describes the number of stem cells that have divided *j-1* times and entered division at time *t*. After division a fraction of *2a_1,j-1_(t)p_1,j-1_(t)c_1,j-1_(t)* belongs to the stem cells that have undergone *j* divisions. The remaining *2(1-a_1,j-1_(t))p_1,j-1_(t)c_1,j-1_(t)* progeny cells belong to *c_2,j_*, i. e., to the class of cells of the second stage of differentiation that have performed *j* divisions. Summarizing, we obtain the following equation, $\frac{dc_{1,j}(t)}{dt}=2a_{1,j-1}(t)p_{1,j-1}(t)c_{1,j-1}(t)-(d_{1,j}(t)+p_{1,j}(t))c_{1,j}(t),$ *0 < j < m*.

Modeling of mature cells. Since cells of the subpopulation *c_1,m_* do not divide (i.e., p_1,m _= 0), there exists only the influx due to division of cells of the population *c_1,m-1_* and the out flux due to death. The corresponding equation reads,

$\frac{dc_{1,m}(t)}{dt}=2a_{1,m-1}(t)p_{1,m-1}(t)c_{1,m-1}(t)-(d_{1,m}(t)+p_{1,m}(t))c_{1,m}(t),$

Modeling of all other maturation steps. In a similar way, for the cells of *i*^th^ stage of differentiation with 0<i<n, we obtain _$\frac{dc_{i,0}(t)}{dt}=(d_{i,0}(t)+p_{i,0}(t))c_{i,0}(t)$_, *0 < i < n*.

Let *0 < j < n*. After division the number of _$2a_{i,j}(t)p_{i,j}(t)c_{i,j}(t)$_ cells belong to the subpopulation *c_i,j+1_* (self-renewing cells at the *i* stage of differentiation) and _$2(1-a_{i,j}(t))p_{i,j}(t)c_{i,j}(t)$_ cells belong to the subpopulation *c_i+1,j+1_* (differentiating cells). Influx to *c_i,j_* from *c_i,j-1_* due to self-renewal is given by _$2a_{i,j-1}(t)p_{i,j-1}(t)c_{i,j-1}(t)$_ and influx from *c_i-1,j-1_* due to differentiation is given by _$2(1-a_{i-1,j-1}(t))p_{i-1,j-1}(t)c_{i-1,j-1}(t)$_. Therefore, we obtain for *0 < i < n*, *0 < j < m*, _$\begin{array}{l} \frac{dc_{i,j}(t)}{dt}=2a_{i,j-1}(t)p_{i,j-1}(t)c_{i,j-1}(t)+2(1-a_{i-1,j-1}(t))p_{i-1,j-1}\\ (t)c_{i-1,j-1}(t)-(d_{i,j}(t)+p_{i,j}(t))c_{i,j}(t), \end{array}$_

Since cells of subpopulation *c_i,m_* do not divide, the corresponding equation reads _$\begin{array}{l} \frac{dc_{i,m}(t)}{dt}=2a_{i,m-1}(t)p_{i,m-1}(t)c_{i,m-1}(t)+2(1-a_{i-1,m-1}(t))\\ p_{i-1,m-1}(t)c_{i-1,m-1}(t)-d_{i,m}(t)c_{i,m}(t), \end{array}$_ *0 < i < n*.

Since mature cells are post-mitotic, there exists no out flux due to division in the subpopulations *c_n,j_*, _0≤j≤m_. Therefore, _$\frac{dc_{n,0}(t)}{dt}=-d_{n,0}(t)c_{n,0}(t),$_ _$\begin{array}{l} \frac{dc_{n,j}(t)}{dt}=2(1-a_{n-1,j-1}(t))p_{n-1,j-1}(t)c_{n-1,j-1}(t)-d_{n,j}\\ (t)c_{n,j}(t), \end{array}$__0<j<m_.

Modeling of feedback regulation. As in the DCF model, we assume that hematopoiesis is regulated by extra-cellular signaling molecules, such as cytokines. The level of the signal depends on the level of mature cells, and is modeled using the equation

_$s(t)=\frac{1}{\left(1+kC_{n}(t)\right)}=\frac{1}{\left(1+k\sum_{0\leq j\leq m}c_{n,j}(t)\right)}$_

This dependence can be justified using a quasi-steady state approximation of the plausible dynamics of the cytokine molecules, [23]. The quasi-steady state approximation is based on the assumption that cytokine metabolism takes place on a different (faster) time scale than cell cycle. The above expression reflects the heuristic assumption that signal intensity achieves its maximum under absence of mature cells and decreases asymptotically to zero if the number of mature cells increases. The signal intensity satisfies is _0<s≤1_. We have previously demonstrated that the regulation of hematopoiesis is much more efficient (and can be achieved in the clinically relevant time scale) only if the feedback mechanism regulates the fraction of self-renewal and differentiation [23]. Regulation of the proliferation rate is not sufficient for that purpose. Therefore, in the reminder of this paper we assume that cell parameters depend on *s* in the following manner: _$p_{i,j}(t)$_ is constant in time, i.e., _$p_{i,j}(t)={\bar{p}}_{i,j}$_, and _$a_{i,j}(t)={\bar{a}}_{i,j}s(t)={\bar{a}}_{i,j}s(C_{n}(t))$_, where _${\bar{p}}_{i,j}$_ and _${\bar{a}}_{i,j}$_ are non-negative constants. Death rates _$d_{i,j}$_ are assumed to be constant in time. In numerical simulations *k *was set as 1.6 x 10^-10^ in analogy to our previous work [23].

Model Equations. The complete model is given by the following system of ODEs.

_$\frac{dc_{1,0}}{dt}=-(d_{1,0}+{\bar{p}}_{1,0})c_{1,0},$_

_$\frac{dc_{1,j}}{dt}=2{\bar{a}}_{1,j-1}s(C_{n}){\bar{p}}_{1,j-1}c_{1,j-1}-(d_{1,j}+{\bar{p}}_{1,j})c_{1,j},$_ for _0<j<m_,

_$\frac{dc_{1,m}}{dt}=2{\bar{a}}_{1,m-1}s(C_{n}){\bar{p}}_{1,m-1}c_{1,m-1}-d_{1,m}c_{1,m},$_

_$\frac{dc_{i,0}}{dt}=-(d_{i,0}+{\bar{p}}_{i,0})c_{i,0},$_for _1<i<n_,

_$\begin{array}{l} \frac{dc_{i,j}}{dt}=2{\bar{a}}_{i,j-1}s(C_{n}){\bar{p}}_{i,j-1}c_{i,j-1}+2(1-{\bar{a}}_{i-1,j-1}s(C_{n})){\bar{p}}_{i-1,j-1}c_{i-1,j-1}\\ -(d_{i,j}+{\bar{p}}_{i,j})c_{i,j}, \end{array}$_for _0<j<m_ and _1<i<n_,

_$\begin{array}{l} \frac{dc_{i,m}}{dt}=2{\bar{a}}_{i,m-1}s(C_{n}){\bar{p}}_{i,m-1}c_{i,m-1}+2(1-{\bar{a}}_{i-1,m-1}s(C)){\bar{p}}_{i-1,m-1}\\ c_{i-1,m-1}-d_{i,m}c_{i,m}, \end{array}$_for _1<i<n_,

_$\frac{dc_{n,0}}{dt}=-d_{n,0}c_{n,0},$_

_$\frac{dc_{n,j}}{dt}=2(1-{\bar{a}}_{i-1,j-1}s(C_{n})){\bar{p}}_{i-1,j-1}c_{n-1,j-1}-d_{n,j}c_{n,j},$_for _0<j≤m_,

with _$s(C_{n})=\frac{1}{\left(1+kC_{n}\right)}=\frac{1}{\left(1+k\sum_{0\leq j\leq m}c_{n,j}\right)}$_.
